# Assessment of genotype imputation methods

**DOI:** 10.1186/1753-6561-3-s7-s5

**Published:** 2009-12-15

**Authors:** Joanna M Biernacka, Rui Tang, Jia Li, Shannon K McDonnell, Kari G Rabe, Jason P Sinnwell, David N Rider, Mariza de Andrade, Ellen L Goode, Brooke L Fridley

**Affiliations:** 1 Department of Health Sciences Research, Mayo Clinic, 200 First Street Southwest, Rochester, MN 55905 USA

## Abstract

Several methods have been proposed to impute genotypes at untyped markers using observed genotypes and genetic data from a reference panel. We used the Genetic Analysis Workshop 16 rheumatoid arthritis case-control dataset to compare the performance of four of these imputation methods: IMPUTE, MACH, PLINK, and fastPHASE. We compared the methods' imputation error rates and performance of association tests using the imputed data, in the context of imputing completely untyped markers as well as imputing missing genotypes to combine two datasets genotyped at different sets of markers. As expected, all methods performed better for single-nucleotide polymorphisms (SNPs) in high linkage disequilibrium with genotyped SNPs. However, MACH and IMPUTE generated lower imputation error rates than fastPHASE and PLINK. Association tests based on allele "dosage" from MACH and tests based on the posterior probabilities from IMPUTE provided results closest to those based on complete data. However, in both situations, none of the imputation-based tests provide the same level of evidence of association as the complete data at SNPs strongly associated with disease.

## Background

Indirect association as a result of linkage disequilibrium (LD) is a key factor in genetic association studies. Because of LD, a disease-susceptibility single-nucleotide polymorphism (SNP) need not be genotyped, as long as it is tagged by a SNP or set of SNPs that are genotyped. This concept has been further exploited by the introduction of methods to impute missing genotypes at untyped markers, based on known genotypes at typed markers and information about LD within the region from a reference panel [1-4]. Such imputation methods can also be applied in the context of combining data across studies with different sets of correlated SNPs genotyped in different studies.

Two recent studies compared imputation accuracy of several methods [5,6]; however, these studies did not assess performance of association tests based on the imputed genotypes. In this paper, we compare the performance of several imputation methods when combining two datasets that have been genotyped at different sets of markers or when completely missing (i.e., "untyped") markers are analyzed. Four commonly used software packages were evaluated: IMPUTE [2], MACH [4], PLINK [7], and fastPHASE [8]. Imputation error rates and performance of association tests using the imputed data were compared. The Genetic Analysis Workshop (GAW) 16 Problem 1 dataset provided by the North American Rheumatoid Arthritis Consortium (NARAC) was used.

## Methods

The NARAC data consisted of 868 cases of rheumatoid arthritis (RA) and 1194 controls genotyped on the 550 k Illumina SNP chip. Four regions were selected on chromosome 1, each consisting of 30 consecutive SNPs, representing regions with disease association (PTPN22 [9,10] and PADI4 [11,12]) and without disease association, and with high or low LD. SNPs deviating from Hardy-Weinberg equilibrium (HWE) (*p *< 0.001) or with call rates below 95% were removed before analysis.

Two scenarios were considered: 1) imputation of "untyped" markers and 2) imputation to combine two datasets.

### Scenario 1

A set of genotyped SNPs were removed completely and subsequently imputed for all subjects. LD plots for the regions as well as a list of removed SNPs are provided by Fridley et al. in this volume [13]. For null regions 1 and 2, seven and eight SNPs were removed, respectively. For the PTPN22 region, two datasets were created with four SNPs excluded in addition to either the most strongly associated SNP (rs2476601) or the two SNPs flanking rs2476601. A similar approach was taken for the PADI4 region, with rs6683201 or the two SNPs flanking rs6683201 removed in addition to five other SNPs.

### Scenario 2

To represent the combined analysis of data from two studies, cases and controls were randomly assigned to two study populations, resulting in 434 cases and 597 controls per group. Genotypes at 10 randomly selected SNPs from each region were removed for all individuals in the first group. A second non-overlapping set of 10 random SNPs were deleted in the second group. Thus, in each region, 10 SNPs were genotyped in both cohorts, while 10 were genotyped only in cohort 1 and were imputed in cohort 2, and 10 were genotyped in cohort 2 and imputed in cohort 1.

Imputation was performed using IMPUTE v 0.4.1 [2], MACH v 1.0.16 [4], fastPHASE v 1.2.3 [8], and PLINK v 0.99 [7]. Haplotypes of the 60 HapMap CEU founders were used as the reference data to run IMPUTE, MACH, and PLINK for scenarios 1 and 2, and to run fastPHASE for scenario 1. For fastPHASE, under scenario 2, only the samples from the NARAC data were used. Programs were run with default options, except to ensure convergence of MACH, each dataset was run with 150 iterations ("--rounds 150"option). In addition the option "--dose" was used with MACH. For imputation of untyped SNPs (scenario 1), the IMPUTE options "-exclude_SNPs file-impute_excluded" were used, while for imputation under scenario 2 the "-pgs" option was used. Full details of the commands used may be obtained from the authors by request.

Our assessment of error rates focused on the proportion of incorrect genotypes obtained by imputing the most likely genotype for each missing value, regardless of the confidence in the imputation. Associations were assessed assuming log-additive allelic effects on RA risk. *p*-Values were calculated using the complete data and each set of imputed data. In addition, for scenario 2, association analyses using the "non-missing data" (genotypes available for only one group) were performed. Association tests based on imputed data used "allele dose" from MACH (the estimated number of minor alleles ranging from 0 to 2), the most likely genotypes imputed using fastPHASE and PLINK, and the posterior probabilities from IMPUTE. For IMPUTE, association tests were performed using the accompanying program SNPTEST, with the "-proper-frequentist 1" options.

## Results

### Error rates

**Figure 1:**
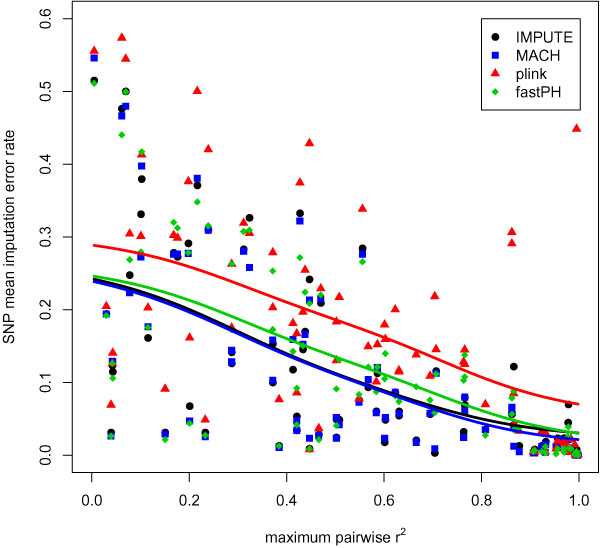
**Imputation error rates decline with increasing LD (scenario 2)**.

Overall, IMPUTE and MACH performed similarly and outperformed PLINK and fastPHASE. Table 1 shows error rates based on imputation of the most likely genotype for each missing value overall, by region, and by maximum pairwise LD. As expected, imputing genotypes at SNPs that are in strong LD with genotyped markers is much more likely to produce correct genotypes. Figure 1 demonstrates this dependence of error rates on LD, using results from scenario 2. Similar results were obtained for scenario 1.

**Table 1:** Mean error rates by imputation method and scenario

		IMPUTE	MACH	PLINK	fastPHASE
**Scenario 1: Imputation of untyped SNPs**					

Overall		0.112	0.114	0.142	0.135
		
By region^a^	null1	0.251	0.251	0.284	0.271
	null2	0.066	0.066	0.090	0.085
	*PADI4*-1	0.083	0.092	0.131	0.111
	*PADI4*-2	0.106	0.109	0.144	0.162
	*PTPN22*-1	0.099	0.098	0.122	0.107
	*PTPN22*-2	0.061	0.059	0.069	0.058
		
By max pairwise	*r*^2 ^< 0.5	0.208	0.212	0.245	0.248
LD	*r*^2 ^≥ 0.5	0.030	0.030	0.053	0.038

**Scenario 2: Imputation to combine two datasets**					
Overall		0.116	0.112	0.173	0.127
By region^a^	null1	0.206	0.201	0.250	0.218
	null2	0.123	0.122	0.175	0.139
	*PADI4*	0.079	0.069	0.145	0.097
	*PTPN22*	0.055	0.053	0.121	0.053
By max pairwise	*r*^2 ^< 0.5	0.200	0.197	0.256	0.211
LD	*r*^2 ^≥ 0.5	0.046	0.041	0.105	0.059

^a^In Scenario 1, for regions *PADI4-1 *and *PTPN22-1*, the most strongly associated SNP was removed and imputed, while for regions *PADI4-2 *and *PTPN22-2*, the two SNPs flanking the most strongly associated SNP were imputed, in addition to other SNPs as described in the methods.

### Association testing

**Figure 2:**
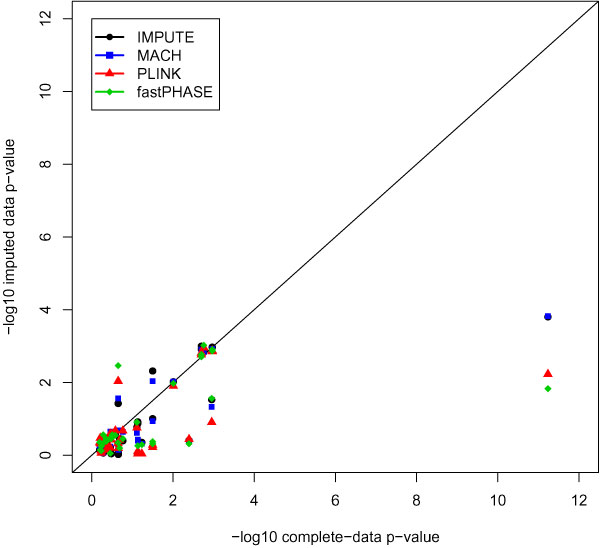
**Comparison of association test results (-log10(*p*-value)) based on complete data with tests based on imputed data under scenario 1 (imputation of untyped markers)**.

**Figure 3:**
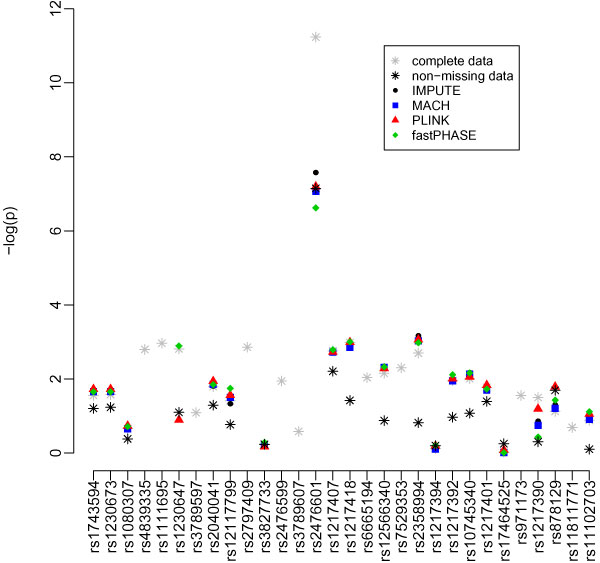
**Association test results (-log10(*p*-value)) based on different imputation methods in the PTPN22 region under scenario 2 (imputation to combine two datasets)**.

Results of association tests based on imputed data are summarized in Table 2 and Figures 2 and 3. Under scenario 1, -log10(*p*-value) for association tests using PLINK-imputed data showed the largest deviation from the complete data -log10(*p*-value). However, with respect to significance testing, the overall performance of the four methods was similar. Performance of these association tests was quite variable between SNPs, as indicated by the large standard deviations in the difference of the -log10(*p*-value).

**Table 2:** Mean (SD) difference^a ^in -log10(*p*-value) based on a test of association using complete data and a test of association using the imputed data

	Scenario 1	Scenario 2
IMPUTE	0.352 (1.26)	0.078 (0.493)
MACH	0.363 (1.27)	0.093 (0.543)
PLINK	0.509 (1.55)	0.054 (0.617)
fastPHASE	0.483 (1.70)	0.046 (0.633)

^a^Difference = (imputed data -log10(*p*-value)) - (complete data -log10(*p*-value))

Comparison of *p*-values from association tests based on the original (complete) data with those that use the imputed data reveals that for SNPs with small association *p*-values, the imputed-data *p*-value tends to be larger than the complete-data *p*-value, consistent with loss of power. This is especially evident at SNP rs24776601 in *PTPN22*, which is strongly associated with RA in the complete data. At this SNP, MACH and IMPUTE provided strongest evidence of association when it was assumed that the SNP had not been genotyped at all (Figure 2), while IMPUTE calculated to the smallest *p*-value when it was assumed that the SNP had been genotyped for half the subjects (Figure 3). In both situations, all imputation-based tests provided substantially less evidence of association than the complete data.

## Discussion

We compared the performance of four commonly used packages for imputation of missing genotype data as well as subsequent tests of association. A key disadvantage of fastPHASE is that it only provides the most likely genotype, while MACH provides an estimate of allele dose, and IMPUTE and PLINK provide estimates of posterior probabilities of all possible genotypes. In agreement with published studies [5,6], when imputing the most likely genotype for each missing value, using MACH and IMPUTE generated lower overall error rates than the other approaches. As expected, imputation was more accurate for SNPs in higher LD with genotyped SNPs. Our method of calculating the error rate did not take into account whether one or two of the alleles are incorrectly imputed. A measure of imputation accuracy that reflects the number of correctly imputed alleles, or uses the posterior probabilities of possible genotypes, could be considered.

On average, association tests based on imputed data gave similar results to the test based on the complete ("unknown") data. However, at the strongest association peak, the imputation-based tests were much less significant than the complete-data test, indicating that using imputation methods followed by association testing can severely underestimate significance at association peaks. This finding may be partially due to the fact that the reference haplotypes used for imputation are representative of a population-based sample that is comparable to the control sample. Dense genotyping of a subset of cases and controls from a given study and use of the resulting haplotypes as the reference data may improve the power of association tests based on imputed data. Further investigation of such an approach is warranted. Although imputation-based tests can underestimate the significance at strongly associated SNPs, they can also lead to results more significant than tests for nearby markers that were genotyped and are indirectly associated with the trait. As with any imputation-based analysis, such results should be interpreted cautiously and the region should be further investigated.

## Conclusion

All methods performed well for SNPs in high LD with genotyped SNPs. However, MACH and IMPUTE generated lower overall imputation error rates and more reliable association test results than fastPHASE and PLINK. Further investigation of the relative merits of using allele doses or posterior genotype probabilities is warranted. The fact that imputation-based tests can severely underestimate significance at strong association peaks warrants caution in using these methods to exclude SNPs from further follow-up.

## List of abbreviations used

GAW: Genetic Analysis Workshop; HWE: Hardy-Weinberg equilibrium; LD: Linkage disequilibrium; NARAC: North American Rheumatoid Arthritis Consortium; RA: Rheumatoid arthritis; SNP: Single-nucleotide polymorphism.

## Competing interests

The authors declare that they have no competing interests.

## Authors' contributions

The study was conceived by BLF. BLF, JMB, ELG, SKM, KRG, and MdA contributed to the conception and design of the study and interpretation of the results. RT, JL, SKM, KGR, JPS, DNR, and BLF ran the analyses. JMB summarized the results and created figures, and JMB, BLF, and ELG drafted the manuscript, which was revised by SKM and KGR. All authors read and approved the final manuscript.
